# Acetylation of insulin receptor substrate-1 is permissive for tyrosine phosphorylation

**DOI:** 10.1186/1741-7007-2-23

**Published:** 2004-11-02

**Authors:** Christina Kaiser, Stephen R James

**Affiliations:** 1 Section of Cell Biology, Department of Biology, Biovitrum AB, SE-112 76, Stockholm, Sweden

## Abstract

**Background:**

Insulin receptor substrate (IRS) proteins are key moderators of insulin action. Their specific regulation determines downstream protein-protein interactions and confers specificity on growth factor signalling. Regulatory mechanisms that have been identified include phosphorylation of IRS proteins on tyrosine and serine residues and ubiquitination of lysine residues. This study investigated other potential molecular mechanisms of IRS-1 regulation.

**Results:**

Using the sos recruitment yeast two-hybrid system we found that IRS-1 and histone deacetylase 2 (HDAC2) interact in the cytoplasmic compartment of yeast cells. The interaction mapped to the C-terminus of IRS-1 and was confirmed through co-immunoprecipitation *in vitro *of recombinant IRS-1 and HDAC2. HDAC2 bound to IRS-1 in mammalian cells treated with phorbol ester or after prolonged treatment with insulin/IGF-1 and also in the livers of ob/ob mice but not PTP1B knockout mice. Thus, the association occurs under conditions of compromised insulin signalling. We found that IRS-1 is an acetylated protein, of which the acetylation is increased by treatment of cells with Trichostatin A (TSA), an inhibitor of HDAC activity. TSA-induced increases in acetylation of IRS-1 were concomitant with increases in tyrosine phosphorylation in response to insulin. These effects were confirmed using RNA interference against HDAC2, indicating that HDAC2 specifically prevents phosphorylation of IRS-1 by the insulin receptor.

**Conclusions:**

Our results show that IRS-1 is an acetylated protein, a post-translational modification that has not been previously described. Acetylation of IRS-1 is permissive for tyrosine phosphorylation and facilitates insulin-stimulated signal transduction. Specific inhibition of HDAC2 may increase insulin sensitivity in otherwise insulin resistant conditions.

## Background

The insulin receptor substrate (IRS) proteins represent key elements in insulin and insulin-like growth factor (IGF) actions, transducing pleiotropic effects on cellular function and regulating processes such as metabolism, growth, cell differentiation and survival [1]. At least four members (IRS 1–4) have been identified that differ with regard to tissue distribution, subcellular localization, developmental expression, binding to the insulin receptor, and interaction with Src homology 2 (SH2) domains. They are all structurally characterised by N-terminal pleckstrin-homology (PH) and phosphotyrosine-binding (PTB) domains, which are required for coupling to the activated insulin/IGF receptors, and a C-terminal region with multiple sites for tyrosine phosphorylation by the receptors. IRS proteins thus act as molecular adapters in recruiting, *inter alia*, a number of SH2-containing proteins binding to specific phosphorylated tyrosine residues. This leads to activation of different intracellular cascades [2], one of which is the PI 3-kinase signalling cascade implicated in mediating the metabolic effects of insulin [3].

The best-substantiated post-translational modification of IRS proteins, in addition to tyrosine phosphorylation, is phosphorylation of specific serine residues. Phosphorylation on these residues is associated both with inhibition of insulin-induced tyrosine phosphorylation of IRS proteins and with facilitation of the effects of insulin [4]. Phosphorylation catalysed by protein kinase C (PKC) isozymes [5,6], c-Jun N-terminal kinase (JNK) [7], inhibitor κB kinase (IKK) isozymes [8], mitogen activated protein kinases (MAPK) [9] and the mammalian target of rapamycin (mTOR) [10] are all associated with reducing the ability of insulin to stimulate tyrosine phosphorylation of IRS proteins and therefore may be part of the physiological and pathophysiological negative regulation of insulin signalling through the IRS pathway. Specific mechanisms explaining why serine phosphorylation leads to reduced tyrosine phosphorylation have not been completely identified, but candidates for this are reduced interaction of IRS proteins with the insulin receptor [11] and increased degradation of IRS [12,13]. Furthermore, phosphorylation of different residues can lead to different effects. Thus, phosphorylation of serine 307 in rat IRS-1 (serine 312 in human IRS-1) is associated with reduced insulin signalling [7] whilst phosphorylation of serine 302 has recently been suggested to facilitate insulin signalling [14], although this has been contested [15].

In addition to phosphorylation of different amino acid residues, insulin signalling through IRS proteins has been shown to be regulated by at least two other mechanisms. Prolonged signal transduction via phosphoinositide 3-kinase (PI3K), which generates the lipid second messenger phosphatidylinositol 3,4,5-trisphosphate, has been shown to induce a state of insulin resistance in cells [16], in part through degradation of IRS-1 [17]. Thus, insulin signalling can be negatively regulated through modulation of IRS concentrations in cells, via degradation of the proteins in the proteasomal pathway [18-20]. The mechanism by which IRS proteins are degraded by the proteasome is not completely understood, but the N-terminal PH and PTB domains are required [21]. In addition, the sub-cellular localisation of IRS proteins may be important for appropriate insulin signalling. The sub-cellular localisation is not absolutely defined, with various lines of evidence pointing to potential places in the cell where the proteins can be found. In addition to the plasma membrane, IRS proteins have been associated with high-density pellets [22] implicating association with the cytoskeleton and recently also with the nucleus [23,24]. Thus, IRS proteins may be located to different parts of the cell where they carry out different functions.

Multiple histone acetyltransferases (HATs) and histone deacetylases (HDACs) control the state of histone acetylation and hence play a regulatory role in modulating the structure and function of chromatin [25]. About 20 HATs have been detected to date, grouped in three different classes on the basis of structural properties. They all have one structural motif in common, the so-called A-motif responsible for acetyl CoA recognition [25]. Several HATs also have non-histone substrates but it is not yet possible to identify putative acetylation sites within a protein simply by sequence analysis. Generally, acetylation affects DNA-binding, protein-protein interactions, protein stability, and protein localization [26]. The acetyl-mediated signals are reversed by HDACs that counteract the effects of HATs by deacetylating lysine residues on histone tails. In higher eukaryotes, HDACs can be subdivided into three distinct groups known as classes I, II, III, according to similarities of their sequences to those of yeast founding members [27]. To date, four enzymes, HDAC1, 2, 3 and 8, are the known members of class I deacetylases [28,29]. HDAC1 and 2 are the best characterised, and are chief constituents of the multiprotein transcriptional-repression complex Sin3/HDAC and the nucleosome remodelling deacetylase NuRD/Mi2/NRD complex [30]. Complexes that contain class I HDACs bind to numerous transcription factors, either directly, or indirectly through the nuclear-hormone corepressors NCOR and SMRT (silencing mediator for retinoid and thyroid hormone receptors). Although all class I and II HDACs can deacetylate histone tails, other cellular proteins can be specifically targeted by different HDACs as well, such as α-tubulin and importin-α [31]. Recent developments have shown that the class I enzymes are regulated by phosphorylation, by casein kinase II amongst others, which increases activity [32-34]. The fact that class II enzymes are phosphorylated has been known for longer, a reaction which is associated with re-localization of the enzymes to the cytoplasm through interactions with 14-3-3 proteins [35].

We now demonstrate that HDAC2 interacts with IRS-1 under conditions when the ability of cells to respond to insulin is compromised. As such, this interaction may constitute a new component of the negative regulation of IRS protein function. We also show that IRS-1 is acetylated, and that augmenting the acetylation level by treating cells with Trichostatin A (TSA, a non-specific inhibitor of HDACs) or with short inhibitory RNA oligonucleotides against HDAC2 partially restores normal responsiveness to insulin.

## Results and discussion

### Interaction between IRS-1 and HDAC2

**Figure 1:**
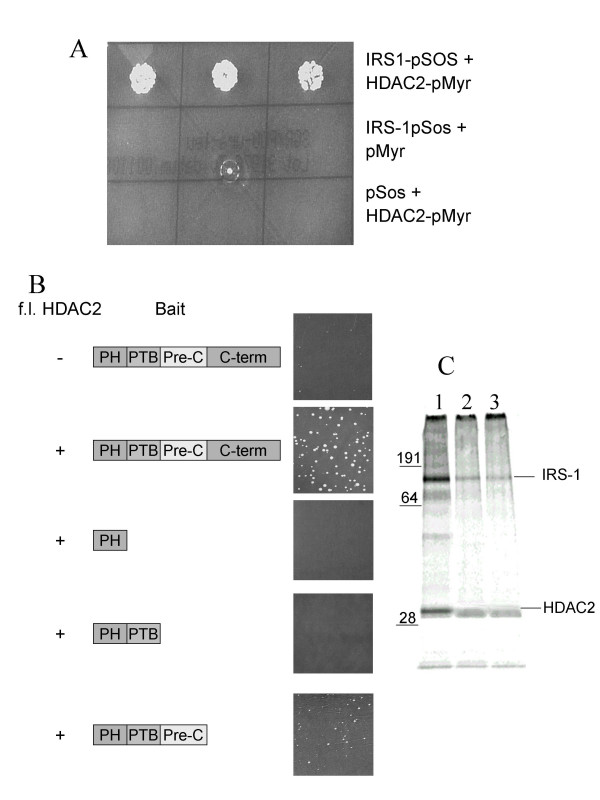
**Analysis of the interaction between IRS-1 and HDAC2 *in vitro***. **A**: A human foetal brain plasmid cDNA library contained in the p-myr vector was transformed into yeast cdc25 h cells containing full length IRS-1 in the pSos vector. Cell growth at the non-permissive temperature (37°C) on galactose medium (GAL 1 promoter in the library vector) was observed only in the presence of both IRS-1 and HDAC2 from the library from two independent transformants, which both encoded human HDAC2. **B**: Full length HDAC2 and different sub-cloned domains of IRS- 1 were used in the Matchmaker-3 yeast two-hybrid system to map the interaction between HDAC2 and IRS-1. Growth on medium lacking two nutritional markers was analysed to confirm interactions between predator and prey. **C**: Recombinant human IRS-1 and HDAC2 were transcribed and translated *in vitro *individually using a rabbit reticulocyte lysate as described in the Methods section. Proteins were then mixed and IRS-1 was immunoprecipitated from the solution (lane 1). As controls for the immunoprecipitation, the IRS-1 antibody was denatured by boiling prior to immunoprecipitation (lane 2) or all antibodies were omitted and beads alone were used (lane 3). Proteins were resolved by SDS-PAGE, the gel was dried and immunoprecipitated proteins were analysed by phosphorimagery. Protein bands matched IRS-1 (approx 160 kD) and truncated HDAC2 (approx. 35 kD).

In an attempt to elucidate the regulation of IRS-1, we investigated inter-molecular interactions between IRS-1 and potential binding partners using yeast two-hybrid screening through a human foetal brain plasmid cDNA library. The system we used was the sos recruitment system as described in the Methods section, which displays protein-protein interactions in the cytoplasm of yeast cells. In these experiments, full length human IRS-1 was used as bait. Two independent transformants from a screen of 4 × 10^5 ^cDNAs encoded the N-terminal portion of a 488 amino acid protein identified as histone deacetylase 2 (HDAC2, Figure 1A).

To map the interaction site of HDAC2 on IRS-1 we used a GAL4-based yeast two-hybrid system, where interactions take place in the nucleus of the yeast cell. Cells were transformed with vectors encoding full length HDAC2 and different truncation mutants of IRS-1. The truncations of IRS-1 that were used were the PH domain (residues 1–155), the PH-PTB domains (residues 1–578) and the PH-PTB-pre-C-terminal domains (residues 1–895). Using growth of yeast cells on selective medium as a readout for interaction between HDAC2 and IRS-1 showed that the interaction requires the C-terminal portion of the IRS-1 protein (Figure 1B). In order to confirm the interaction further *in vitro*, we used a coupled *in vitro *transcription/translation system in which full length IRS-1 and the HDAC2 N-terminal portion from the initial yeast two-hybrid screen were transcribed and translated in the presence of S35 methionine. IRS-1 was subsequently immunoprecipitated from the mixture and the proteins were resolved by SDS-PAGE. Gels were then subjected to autoradiography. Results showed that two radioactive protein bands were visible in the IRS-1 immunoprecipitates (Figure 1C) and their molecular weights corresponded to those of full length IRS-1 (approx 160 kD) and truncated HDAC2 (approx 35 kD). When the IRS-1 antibody was boiled prior to immunoprecipitation (Figure 1C lane 2) or omitted (Figure 1C lane 3), no radioactive proteins were observed, indicating that the interaction between the two proteins is specific and not due to non-specific interactions with immunoglobulins or beads. Thus, IRS-1 and HDAC2 proteins are able to interact with each other in cell-free systems.

**Figure 2:**
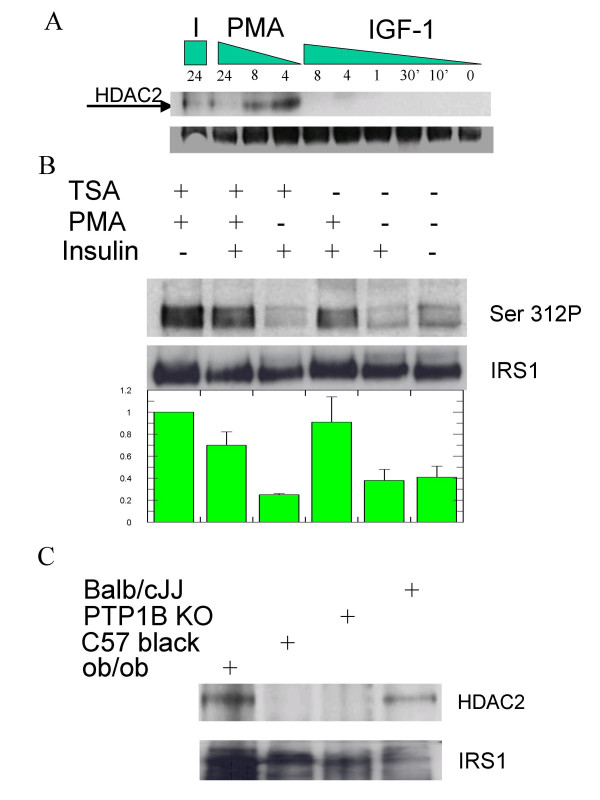
**Analysis of conditions for interaction between IRS-1 and HDAC2 in mammalian cells and tissues. A**: MCF-7 human breast carcinoma cells were treated with 10 ng/ml IGF-1 ("I") or 10 ng/ml PMA for the indicated times. IRS-1 was immunoprecipitated from cell lysates and western blots were analysed for co-immunoprecipitation of HDAC2. The upper gel shows presence of HDAC2 whilst the lower gel is a loading control for IRS-1. The data are representative of multiple experiments. Similar data are obtained if IGF-1 is exchanged for 100 nM insulin. **B**: MCF-7 cells were treated PMA (10 ng/ml) or TSA (150 ng/ml) for 4 hours prior to stimulation with or without insulin (100 nM) for 10 minutes, lysis and immunoprecipitation of IRS-1. Western blots were probed for the presence of phospho-serine 312 in IRS-1 (upper gel) and IRS-1 (lower gel). The histogram shows the means ± range for results for serine 312 phosphorylation from two independent experiments, normalised to the maximum phosphorylation signal, which was seen in cells treated with PMA and TSA. In separate control experiments, we have seen that the phosphorylation of serine 312 in cells is driven by PMA and that TSA does not contribute to the effect (data not shown). **C**: Liver tissues from ob/ob mice, c57/bl6 mice, PTP1B knockout mice and balb/cjj mice were prepared as described in the Methods section and IRS-1 was immunoprecipitated. Western blots were analysed for the presence of HDAC2 (upper gel) or IRS-1 (lower gel). Each lane is from one mouse and is representative of at least two other animals per group.

To validate the interaction between IRS-1 and HDAC2 further and to ascertain whether IRS-1 and HDAC2 are associated in mammalian systems, we chose to work with MCF-7 cells (a human breast adenocarcinoma cell line), with a high endogenous expression of IRS-1 [36]. The cells were stimulated with IGF-1 or PMA (phorbol myristic acid; a PKC activator known to inhibit growth factor signalling [37]) for different time periods. Immunoprecipitations with IRS-1 antibody revealed that HDAC2 was co-precipitated to a larger extent in PMA-treated cells (Figure 2A). In addition, the interaction between IRS-1 and HDAC2 was more visible in cells under prolonged stimulation with IGF-1. Similar results were obtained during prolonged stimulation with insulin. Considering that the ability of cells to respond to insulin and IGF-1 is reduced after prolonged ligand stimulation or PMA treatment, these data indicate that IRS-1 and HDAC2 associate when responsiveness is low and intracellular serine phosphorylation is increased. Indeed, analysis of serine phosphorylation of IRS-1 after treatment of cells with insulin or phorbol ester showed that PMA treatment caused a significant increase in phosphorylation of IRS1 on serine 312 (equivalent to serine 307 in rat IRS-1), which has been associated with reduced phosphorylation on tyrosine residues by the insulin receptor (Figure 2B lanes 1,2 and 4), whereas insulin stimulation had no effect. In these experiments, cells were stimulated with insulin for 10 minutes and responsiveness was subsequently analysed by measuring tyrosine phosphorylation of IRS-1 (see Figure 4 and discussion below). Responsiveness of the cells to insulin was compromised after PMA treatment, thus confirming the apparent association of IRS-1 with HDAC2 under conditions of reduced cellular sensitivity to insulin.

To assess whether the interaction measured between IRS-1 and HDAC2 *in vitro *as described above occurs *in vivo*, we prepared lysates of liver tissues prepared from different mouse lines. The ob/ob mouse, which lacks functional leptin, was chosen as an insulin resistant animal model, and C57/bl6 was used as its genotype control. A PTP1B knockout mouse [38] was used as an insulin-sensitised animal model and balb/cJJ was used as its genotype control. IRS-1 was immunoprecipitated from liver lysates and western blotted for co-immunoprecipitation of HDAC2. The data showed that whilst a clear interaction between IRS-1 and HDAC2 was seen in livers from ob/ob mice (Figure 2C), no interaction was evident in the C57/bl6 control. In contrast, no interaction was evident in livers of PTP1B knockout animals, whilst the balb/cJJ genotype control demonstrated a measurable interaction. Taken together with the *in vitro *data, these results showed that IRS-1 and HDAC2 are able to interact with each other in the cytoplasmic compartment of cells and that the interaction occurs under conditions of reduced insulin sensitivity, both in mammalian cells and in animals. The cytoplasmic location of the interaction is interesting in view of the fact that HDAC2 is considered to be largely a nuclear protein. In our work with cells and tissues, we have utilised lysis methods that are designed to retain nuclei intact and thereby minimise cross-contamination of compartments [39,40]. Whilst we have not formally excluded the possibility of contamination of cytoplasmic extracts with nuclear lysate, thereby leading to the presence of HDAC2, we feel that the body of evidence indicates that cytoplasmic HDAC2 is interacting with cytoplasmic IRS-1 in our experiments. The yeast two hybid "Sos recruitment system" is built on the rescue of cell growth through the interaction of proteins in the cytoplasm, which is how we detected this interaction. Interestingly, it has recently been shown that histone deacetylase 1, another class I histone deacetylase, which was considered to be exclusively nuclear, is present in a cytoplasmic protein complex by virtue of interaction with a cellular phosphatase complex [41].

### Lysine acetylation of IRS-1 and insulin signal transduction

**Figure 3:**
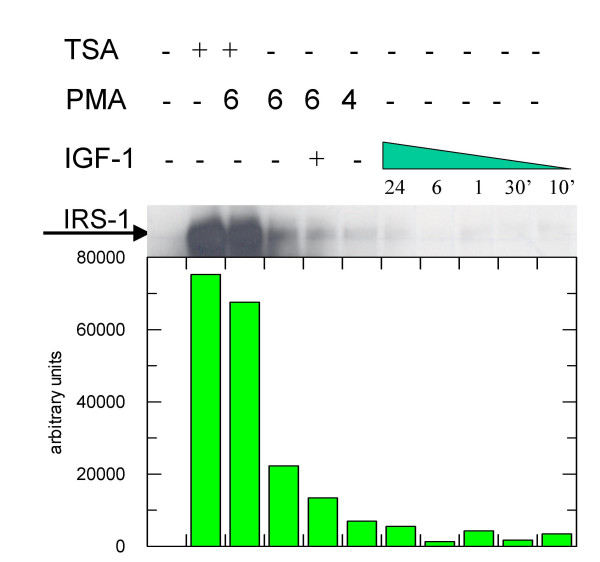
**IRS-1 is a lysine acetylated protein. **MCF-7 cells were treated with IGF-1 (10 ng/ml) for the indicated times, PMA (10 ng/ml) for 4 or 6 hours as indicated or TSA (150 ng/ml) for 4 hours prior to lysis and immunoprecipitation of IRS-1 Western blots were probed for the presence of acetyl lysine. Densitometric analysis of acetylated IRS-1 was performed and is displayed in the histogram. This experiment has been repeated one other time although the effects of TSA on acetylation of IRS-1 have been observed in many other experiments.

The finding that HDAC2 binds to IRS-1 indicated that IRS-1 might be an acetylated protein in which acetylation might be a regulated post-translational modification of the protein. Indeed, the acetyl transferase Tip60 has been reported to bind to the PH domain of IRS-1 [42], suggesting the IRS-1 could be acetylated and deacetylated under different conditions. The lysine-acetylation status of IRS-1 was assessed by western blotting of IRS1 immunoprecipitated from MCF-7 cells after different treatments, using an antibody specific for acetylated lysine. Trichostatin A (TSA), which is a non-selective inhibitor of both class I and class II HDACs [43], was used as a positive control. Basal acetylation of the IRS1 protein was evident in unstimulated cells (Figure 3). Stimulation of cells with IGF-1 did not alter the level of acetylation although the basal signal was low and small effects cannot therefore be ruled out. PMA was also ineffective in altering the basal degree of acetylation of IRS1 whereas treatment of cells with TSA caused a very large increase in signal (Fig. 3). Our data therefore show that IRS-1 protein is acetylated on lysine residues, and the acetylation increases when HDAC activity is generally inhibited. This represents a heretofore-undescribed post-translational modification of IRS1 in addition to tyrosine/serine phosphorylation and ubiquitination previously described. TSA treatment did not induce phosphorylation of IRS1 on serine 312 (Fig 2B lane 3), nor did it modify the increase in serine 312 phosphorylation in the presence of PMA (lanes 1 and 2).

**Figure 4:**
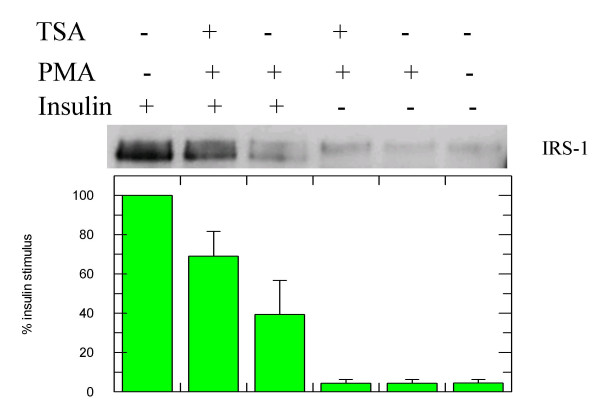
**Increases in IRS-1 lysine acetylation enhance insulin signalling to IRS-1. **MCF-7 cells were treated with PMA and/or TSA for 4 hours (concentrations as in Figure 3) prior to stimulation with 100 nM insulin for 10 minutes. IRS-1 was immunoprecipitated and blotted for the presence of phosphotyrosine. The graph summarises results from 3 experiments (± SEM) normalised to the response to insulin stimulation alone.

The regulation and function of proteins such as sterol regulatory element binding protein 1c (SREBP1c) [44] and p53 [45] has been shown to be altered by changes in acetylation. The alterations in lysine acetylation in IRS-1 induced by TSA raised the possibility that insulin signal transduction may be altered in cells after treatment with this compound. To assess the effects of changes in IRS-1 acetylation on insulin signalling, MCF-7 cells were treated with PMA, TSA and insulin in different combinations and immunoprecipitated IRS-1 protein was immunoblotted for the presence of phosphotyrosine. PMA alone and in combination with TSA did not increase tyrosine phosphorylation of IRS1 above basal, as expected (Fig 4 lanes 4–6). Furthermore, the ability of insulin to induce tyrosine phosphorylation of IRS-1 was reduced by 60% in cells pre-treated with PMA (Fig 4 lane 3) consistent with a state of insulin unresponsiveness. However, pre-treatment with TSA in the presence of PMA reduced this unresponsiveness, increasing insulin-stimulated tyrosine phosphorylation to 70% of control (Fig 4 lane 2). Thus, increases in IRS1 acetylation via TSA-mediated HDAC inhibition were able to restore insulin signalling significantly. This restoration occurred without reducing PMA-induced serine 312 phosphorylation of IRS-1 (Fig 2B lane 2), indicating that acetylation of IRS1 overcomes the inhibitory effects of phosphorylation of serine 312.

**Figure 5:**
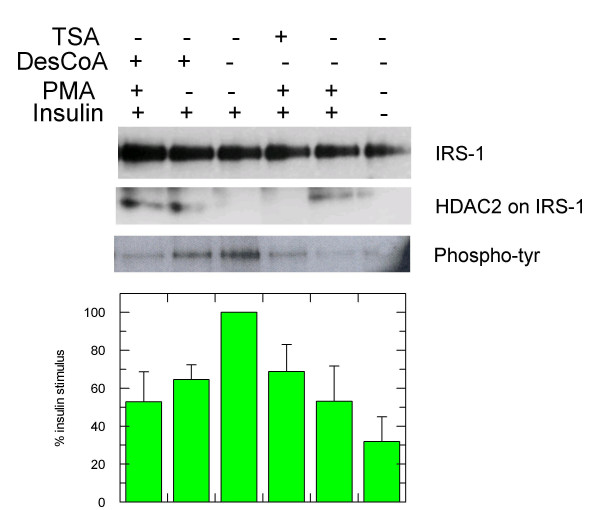
**Inhibition of HAT activity inhibits insulin signalling. **MCF-7 cells were treated with PMA, TSA and DesCoA (10 nM) in the indicated combinations for four hours prior to stimulation with insulin for 10 minutes. IRS-1 was immunoprecipitated and western blotted for the presence of IRS-1 itself, HDAC2 and phosphotyrosine. The graph shows average data for phosphotyrosine from three independent experiments (± SEM) normalised to the response to insulin alone.

To assess the relative roles of altered intracellular protein acetylation and binding of HDAC2 to IRS-1 on insulin signalling, we treated cells with the general HAT inhibitor, desulfo coenzyme A (DesCoA, [46]) and examined HDAC2-IRS-1 interactions and insulin-stimulated tyrosine phosphorylation of IRS-1. The data showed that treatment with DesCoA induced HDAC2 to bind to IRS-1 to a similar extent to phorbol ester, which was coincident with reduced insulin-stimulated tyrosine phosphorylation of IRS-1 (Figure 5). In these experiments, interactions between HDAC2 and IRS-1 were apparently weaker in cells treated in the presence of TSA. This is not a consistent phenomenon, and occurs to varying degrees in our experiments (unpublished data). However, TSA has been reported to break other cellular HDAC-phosphatase complexes [41], so the effect here on HDAC2 and IRS-1 is not unprecedented. Treatment of cells with PMA and DesCoA did not lead to significantly greater effects, indicating that the two compounds share a common mechanism of reducing insulin signalling. Thus, inhibition of intracellular lysine acetylation accompanied by interactions between IRS-1 and HDAC2 leads to compromised insulin signalling, which can be overcome by inhibition of HDAC activity. Casein kinase II is an enzyme that has been shown to regulate the ability of HDAC2 to form oligomeric complexes both positively and negatively [32]. Interestingly, treatment with an inhibitor of casein kinase II (5,6-dichloro-1-β -D-ribofuranosylbenzimidazole) did not induce binding between IRS-1 and HDAC2 (Kaiser & James, unpublished) and had no effect on insulin signalling.

**Figure 6:**
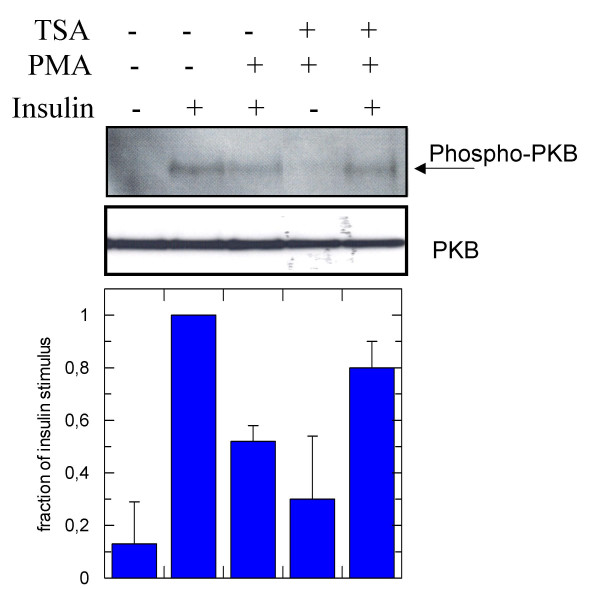
**Distal insulin signalling is enhanced by increased IRS-1 lysine acetylation. **MCF-7 cells were treated with PMA, TSA and insulin as for Figure 4 and lysates were western blotted for the presence of PKB phosphorylated on serine 474. The graph shows average data from three independent experiments (± SEM) normalised to the insulin response alone.

To ascertain if more distal insulin signalling was also enhanced, we examined the activation of protein kinase B (PKB) by western blotting, using an antibody against PKB phosphorylated on serine 474; this phosphorylation is induced in a PI3K-sensitive manner resulting in enhanced protein kinase activity. The data showed that PMA treatment reduced the activation of PKB by 50% (Figure 6 lane 3), whereas with pre-treatment with TSA, the response was 80% of control (Figure 6 lane 5). Thus, TSA-mediated increases in lysine acetylation of IRS-1 led to virtual restoration of PKB activation by insulin in PMA-treated cells. Interestingly, the PKB response in the presence of TSA and PMA (but no insulin, Figure 6 lane 4) showed significantly higher basal activation of PKB than in unstimulated cells. We speculate that this is due to the recently described ability of HDAC inhibitors, including TSA, to activate PKB through an unknown mechanism [47]. Thus, the increased response of cells to insulin in the presence of TSA (Figure 6 lane 5) may represent a summation of the effects of TSA alone and insulin. One candidate mechanism for the reported activation of PKB by HDAC inhibition is via increased acetylation of IRS-1 leading to enhanced basal PI3K activity and enhanced PKB activity. We could not, however, detect increases in basal tyrosine phosphorylation of IRS-1 in the presence of TSA without insulin (Figure 4 lane 4), suggesting that, if this is indeed part of the mechanism of activation of PKB by HDAC inhibition, it is beyond the limits of detection. Such a possibility is not without precedent. We have previously reported the ability of a non-specific protein tyrosine phosphatase inhibitor to increase PI3K-dependent glucose transport in muscle cells in culture without being able to detect changes in basal tyrosine phosphorylation of the insulin receptor or IRS-1 [48]. Thus, it remains possible that HDAC inhibition by TSA leads to enhanced PKB phosphorylation through small changes in IRS-1 phosphorylation.

A major functional response downstream of the PI3K arm of insulin signal transduction is increased glucose transport mediated by the GLUT4 transporter. We sought to examine the effects of TSA on glucose transport in rat L6 myotubes to see if the enhanced insulin signalling mediated by TSA treatment of cells translated into increased glucose uptake. We found that treatment of L6 myotubes with PMA resulted in increased basal glucose transport and had no effect on insulin-stimulated glucose transport (Kaiser & James, unpublished). Such effects are in line with data presented for rat epitrochlearis muscle [49] and indicated that L6 cells do not exhibit a clear insulin-resistance phenotype after PMA treatment, at the level of glucose transport. We also have similar observations in the human neuroblastoma cell line SHSY-5Y, which demonstrates insulin-stimulated glucose uptake [50]. Phorbol ester treatment of these cells increased basal glucose transport but in contrast to data in L6 cells, also inhibited insulin-stimulated glucose transport (Kaiser and James, unpublished). We have therefore not been able to distinguish an effect of TSA on GLUT4-mediated glucose transport owing to the large PMA-stimulated increases in insulin-independent glucose transport (presumably mediated by GLUT1), and are at present analysing other cells for their response to phorbol ester treatment. Interestingly, Takigawa-Imamura et al. [51] recently showed that several HDAC inhibitors increase glucose transport in muscle cells in culture. Although the treatment regimens with these inhibitors in these experiments were chronic, the data show that inhibition of HDAC activity enhances glucose transport. Molecular mechanisms behind this effect could be several, including enhanced insulin signalling through increases in intracellular protein acetylation.

**Figure 7:**
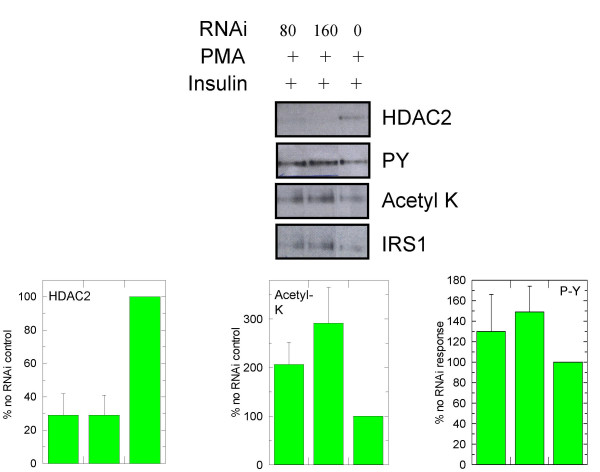
**Specific inhibition of HDAC2 enhances insulin signalling. **MCF-7 cells were transfected with short inhibitory RNA oligonucleotides (80 and 160 pmol as indicated) for four hours and cultured a further 48 hours prior to treatment with PMA (10 ng/ml for 4 hours) and stimulation with insulin (100 nM) for 10 minutes. IRS-1 was immunoprecipitated and western blotted for the presence of HDAC2, phosphotyrosine, acetyl lysine and IRS-1 itself. The graphs show average data (± SEM) for 3–5 independent experiments, normalised to the insulin response alone. P-Y: phosphotyrosine.

TSA is an efficacious inhibitor of all class I and class II HDAC enzymes, with a potency in the low nanomolar range. To ascertain whether specific inhibition of HDAC2 activity is able to enhance insulin signalling in otherwise non-permissive conditions (PMA treatment), we used RNA interference to reduce HDAC2 activity specifically. MCF-7 cells were transiently transfected with a 21 base RNA duplex oligonucleotide against HDAC2 which reduced the HDAC2 protein content of the cells by approximately 70% (Figure 7). This was associated with a greater than three-fold increase in lysine acetylation of IRS-1. Furthermore, insulin-stimulated tyrosine phosphorylation of IRS-1 was increased 1.5-fold in RNAi-treated cells (Figure 7). A second RNAi oligonucleotide against HDAC2 was found to be much less efficient in silencing, exerting no effect on HDAC2 expression at 25 nM (corresponding to 80 pmol, see Methods section). Control experiments with this oligonucleotide at concentrations when HDAC2 expression was unaffected, showed that insulin-stimulated tyrosine phosphorylation of IRS-1 was not affected (data not shown), indicating that specific reductions in HDAC2 after RNAi treatment were the main cause of enhanced insulin signalling and IRS-1 acetylation. These data showed that specific reductions in HDAC2 activity in MCF-7 cells induced similar changes in IRS-1 regulation as treatment with TSA and that HDAC2 is an integral component of phorbol ester-induced insulin unresponsiveness in cells. The increase in lysine acetylation and tyrosine phosphorylation was arguably not as marked in RNAi-treated cells as in cells treated with TSA. An interpretation of these data could be that other members of the HDAC family are also involved in the processes leading to insulin resistance. We have found that HDAC1 does co-immunoprecipitate with IRS-1 from MCF-7 cells but its regulation is different, with no significant changes in the association by prolonged insulin stimulation or by PMA treatment of the cells (Kaiser & James, unpublished) suggesting that whatever the involvement of other HDACs, HDAC2 is central to the observed changes in insulin signalling.

The data we present here imply that treatment of insulin-resistant or diabetic animals with inhibitors of HDAC2 should increase insulin responsiveness. We attempted to assess the effects of TSA on insulin sensitivity in *ob*/*ob *mice. The animals were divided into two groups: vehicle (DMSO) and TSA (0.1 mg/kg) and treated subcutaneously for three days. At the same time as drug injection, all food was withdrawn from the animals and 4 hours later, blood was collected from the tail vein for blood glucose and plasma insulin analysis. On the third day, an insulin tolerance test (ITT) was performed 4 hours after administration of the drug. After 24 hours, fasting blood glucose tended to be lower in treated animals than vehicle controls, but after three days no difference was evident. Furthermore, we were unable to detect a change in insulin sensitivity after drug treatment during the ITT on day 3 (Kaiser, Warpman & James, unpublished). In addition, no changes in lysine acetylation of IRS-1 were observed, indicating that the lack of effect on insulin sensitivity could be due to the inability of TSA to work through the molecular mechanism of increasing IRS-1 acetylation. TSA is rapidly metabolised by liver cells in culture in two stages, initially by reduction to the imide followed by demethylation, leading to inactive metabolites [52]. It is therefore probable that the compound was rapidly metabolised by hepatic phase I metabolic processes in these experiments so that it was unable to exert pharmacodynamic effects on the animals. The poor bioavailability of TSA [53] has led to its discontinuation as a clinical candidate for the treatment of human disease and the possibility of testing the insulin sensitizing effects of HDAC inhibition must await the availability of a drug with better pharmacokinetics. Furthermore, HDACs are not redundant, but have specific expression patterns and functions. Therefore, it is of great importance to develop specific HDAC-inhibitors to be able to assess their respective contributions to increases in insulin sensitivity in vivo.

**Figure 8:**
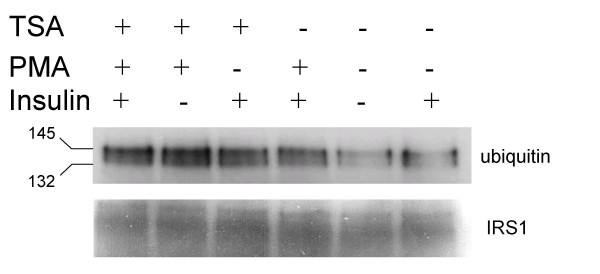
**IRS-1 ubiquitination is not altered by acetylation. **MCF-7 cells were treated with PMA, TSA and insulin as described for Figure 4 and immunoprecipitated IRS-1 was western blotted for the presence of ubiquitin (upper panel) and IRS-1 (lower panel). The molecular mass of the immunoprecipitated ubiquitinated protein and IRS-1 protein is indicated.

The mechanism whereby lysine acetylation of IRS-1 leads to increased tyrosine phosphorylation by the insulin receptor is not known. Time course experiments, in which cells were stimulated with insulin for one to ten minutes, showed that the kinetics of IRS-1 phosphorylation were the same, irrespective of pre-treatment of cells with TSA (Kaiser & James, unpublished). However, the IRS-1 tyrosine phosphorylation signal was greater at all times in cells treated with TSA, suggesting that lysine acetylation of IRS-1 simply increases the amount of phosphorylated IRS-1. It has recently been shown that lysine acetylation protects the transcription factor SREBP1C from ubiquitination and degradation via the proteasomal pathway by competing for the same lysine residues. IRS-1 has also been shown to be degraded via ubiquitination and subsequent proteasomal degradation [21]. We investigated the influence of lysine acetylation of IRS-1 on ubiquitination by blotting immunoprecipitates of IRS-1 from cells for the presence of ubiquitin (Fig 8). The data showed that in the absence of PMA, IRS-1 was only slightly ubiquitinated, whereas in cells treated with PMA, this was markedly increased. The molecular mass of both bands of the IRS-1 doublet increased after PMA treatment, spanning 132 kD to 145 kD, presumably due to the addition of ubiquitin molecules. TSA did not influence PMA-induced ubiquitination of IRS-1. These data therefore indicate that increases in IRS-1 phosphorylation after its lysine acetylation are not the result of increasing the concentration of the protein by preventing its degradation. Interestingly, a protein called PH domain interacting protein (PHIP) was recently described that selectively binds *in vitro *and constitutively associates in cells to the PH-domain of IRS-1 [54]. PHIP is not itself a substrate of the insulin receptor but rather a ligand of the IRS-1 PH-domain that serves to link IRS-1 to the insulin receptor and enhance its phosphorylation. PHIP contains two bromodomains located in tandem in the centre of the molecule [55]. Considering the fact that IRS-1 is acetylated and that bromodomains can interact specifically with acetylated lysine [56], the mode of interaction between PHIP and IRS-1 could be through the bromodomains, providing a molecular mechanism that explains why the increased acetylation of IRS-1, after TSA treatment, is accompanied by a higher level of tyrosine phosphorylation of IRS-1 despite the insulin resistant state. We have sought to test this hypothesis by blotting immunoprecipitates of IRS-1 for PHIP but have been unable to distinguish a specific band corresponding to PHIP using the antibodies that are available commercially.

## Conclusions

In this study, we have identified a previously undescribed interaction between IRS-1 and HDAC2 in the cytosolic compartment of cells. The interaction is observed both *in vitro *and *in vivo *during conditions of compromised insulin signalling, as seen by reductions in insulin-stimulated IRS-1 tyrosine phosphorylation and PKB activation and increased phosphorylation of the negative regulatory phosphorylation site, serine 312. Our data indicate that it is the interaction with HDAC2 itself rather than its catalytic activity that is integral to the insulin unresponsiveness that ensues. Furthermore, our data show that IRS-1 is a lysine-acetylated protein, a previously unidentified post-translational modification of IRS-1, and that increases in the level lysine acetylation of IRS-1 result in improved insulin signal transduction. Increases in IRS-1 acetylation can be achieved pharmacologically (with TSA) or by ablation of HDAC2 specifically by use of RNAi. Out data therefore indicate that a new dimension to the physiology and pathophysiology of insulin sensitivity and insulin resistance involves changes in the degree of lysine acetylation of IRS-1 and that specific small molecule inhibitors of HDAC2 activity could represent novel therapeutics for the treatment of diseases that centre around insulin resistance, such as type 2 diabetes and obesity.

## Methods

### Yeast two hybrid screening

The CytoTrap™ (Stratagene) yeast two-hybrid system was used to discover protein-protein interactions in the cytoplasm of yeast cells. Interactions were detected by recruitment to the cell membrane of the human Sos (hSos) gene product, which activates the Ras pathway. The yeast strain used (cdc25H) harbours a temperature sensitive mutation in the cdc25 gene, the yeast homologue for hSos, which means that the cells can grow at 25°C but not at 37°C unless rescued with a protein-protein interaction. A human foetal brain plasmid cDNA library (Stratagene), harboured in the pMyr vector (with a myristylation signal to direct and anchor proteins in the membrane), was used as "prey" and the sub-cloned full length IRS-1 gene in the pSos vector was used as "bait". When prey and bait proteins interact the hSos is brought into close proximity to Ras and subsequently the yeast survive and are selected by growth at 37°C. The IRS-1/HDAC2 interaction rescued growth at 37°C in this way. The corresponding pMyr yeast plasmid was isolated and co-transformed with the pSos bait construct to perform false positive tests.

HDAC2 was full length cloned using RACE cDNA obtained from human heart tissue together with gene specific primers and the Advantage 2 polymerase mix (Clontech). With the purpose of mapping the interaction site of HDAC2 on IRS-1 we used the Matchmaker 3 yeast two-hybrid system (Clontech). This is a GAL4-based two-hybrid system that provides a transcriptional assay for detecting specific protein-protein interactions in yeast. Two nutritional markers and one enzymatic reporter gene were used to detect interactions. Different domains of IRS-1 (PH domain, residues 1–155, the PH-PTB domains, residues 1–578 and the PH-PTB-pre-C-terminal domains, residues 1–895, obtained by PCR) were sub-cloned into a "bait" vector (pGBKT7), fused to the DNA-binding domain of GAL4. Full length HDAC2 was sub-cloned into the "prey" vector (pGADT7), fused to the activation domain of GAL4. Cell growth on medium lacking the two nutritional markers was used as a readout of the interaction between the predator and prey.

### *In vitro *transcription-translation

In order to confirm the IRS-1/HDAC2 interaction *in vitro*, we used a coupled transcription/translation system (Promega) comprising a rabbit reticulocyte lysate solution with RNA polymerase, nucleotides, salts, a ribonucleoside inhibitor, and [^35^S]-methionine (Amersham Biosciences) to allow detection of translated proteins. Since the prey vector pMyr already contains a T7 promoter, this was used directly in the system. However, the bait vector pSos lacks a T7 promoter and thus the IRS-1 gene was subcloned into a T7-containing vector (pGBKT7; Clontech) to permit transcription. The individually transcribed and translated proteins were mixed and co-immunoprecipitated with anti-IRS-1 antibodies (Upstate Biotechnologies) and subsequently analysed by polyacrylamide gel electrophoresis (4–12%). The gel was dried analysed by phosphorimagery.

### Cell culture

The human breast adenocarcinoma cell line MCF-7 was cultured in a mixed medium of Dulbecco's Modified Eagle Medium with nutrient mixture F12 (Invitrogen) lacking phenol red with 10% Foetal Bovine Serum (Gibco). At near confluency, cells were starved of serum for 16 h and subsequently treated with IGF-1, insulin, PMA (phorbol myristic acid; Sigma) or TSA (Trichostatin A; Sigma), or combinations thereof, for different lengths of time as indicated in individual figures. Cells were harvested in hypotonic cell lysis buffer comprising 20 mM Hepes, pH 7.6, 20% glycerol, 10 mM NaCl, 1.5 mM MgCl_2_, 0.2 mM EDTA, 0.1% NP40, 25 mM NaF, 25 mM β-glycerophosphate, 1 mM DTT, 1 mM Na-orthovanadate and protease inhibitors.

### Western blot assays

Cell lysates were cleared by centrifugation at 16000 g for 10 min at 4°C, and protein content was determined using the Bradford method (BioRad). For immunoprecipitations, matched amounts of protein were incubated with primary antibody (amount used as recommended by the manufacturer or empirically determined) for 2 h at 4°C followed by addition of 20μl of protein A/G agarose suspension (Santa Cruz) for 1 h at 4°C with rotating tube. After washing (3 times with high salt (500 mM NaCl) and twice with isotonic buffer), beads were heated with SDS-PAGE sample buffer for 10 minutes at 70°C and proteins were resolved by 4–12% gradient SDS-PAGE. After blotting, membranes were blocked in 5% non-fat dried milk in Tris-buffered saline containing 0.1% Tween 20 for 1 h prior to addition of the primary antibody. After incubation with secondary horseradish peroxidase-conjugated antibody, protein bands were visualised using enhanced chemiluminescence (ECL-plus detection kit, Amersham Biosciences).

Antibodies used were anti-IRS-1 (Upstate, cat. no 06–248); anti-HDAC2 (Santa Cruz, cat. no. sc-9959 and sc-6296); anti-phosphotyrosine (Santa Cruz, cat. no. sc-7020); anti-acetyl lysine (Cell Signalling, cat. no. 9681); anti-ubiquitin (Santa Cruz, cat. no. sc-6085 and sc-9133); anti-phospho-serine 307 IRS-1 (Upstate, cat. no. 07–247), HRP-conjugated anti-mouse IgG (Amersham Biosciences, cat. no. NA931V); HRP-conjugated anti-goat IgG (Dako cat. no. PO449) and HRP-conjugated anti-rabbit IgG (Upstate, cat. no. 12–348).

### RNA interference

Double stranded RNA duplexes corresponding to amino acids from the C-terminal part of human HDAC2 (5'CAGCUCAGCAACCCCUGAAtt3') were annealed and transfected into human MCF-7 cells (Lipofectamine 2000 from Invitrogen was used as transfection agent): The effect of RNAi on HDAC2 expression and on insulin dependent IRS-1 tyrosine phosphorylation was measured after 48 hours. A second oligonucleotide (5'GGAGCAAAGAAAGCUAGAAtt3') was found to be non-silencing at a dose of 80 pmol, in contrast to the silencing oligonucleotide above, and was used in control experiments showing that no effect on IRS-1 phosphorylation or acetylation was observed (data not shown).

### Animal experiments

Male 8-week old *ob*/*ob *mice were obtained from Bomhultsgard, Denmark and housed according to standard procedures. C57/bl6 genotype control mice were obtained from Scanbur BK AB (Sollentuna, Sweden). PTP1B knockout animals on a balb/cJJ background were purchased from McGill University, Montreal, Canada. Balb/cJJ genotype controls were obtained from Scanbur BK AB. In our hands, balb/cJJ mice are generally a healthy mouse strain that breeds well. In side-by-side experiments, the mice are more insulin sensitive than C57/bl6 mice whilst being less insulin sensitive than the PTP1B knockout animals on the same genetic background. The animals are somewhat smaller than C57/bl6 mice and have a relatively high body fat content.

For compound treatment experiments and insulin tolerance tests, animals were divided into two groups: vehicle (1% DMSO sub-cutaneous injection (s.c.), n = 15 and TSA 0.1 mg/kg s.c., n = 15) and subsequently treated s.c. at 09.00 h for three days. At the same time as injection, all food was withdrawn from the animals. Four hours later, blood was collected from the tail vein for blood glucose and plasma insulin analysis. On the third day, an insulin tolerance test (ITT) was performed 4 h after administration of the drug. ITT: insulin (actrapid 0.5 U/kg) was administered i.p. Following insulin administration, blood samples were collected after 15, 30, 60, 120 and 180 min from the tail vein for glucose analysis. Animals were then sacrificed and livers were dissected and immediately frozen in liquid nitrogen and stored at -70°C. All experiments were performed in accordance with permission from the local Swedish ethics committee and the company Pharmacology ethics review team.

For western blotting of liver proteins, frozen liver was powdered finely under liquid nitrogen using a pestle and mortar pre-cooled to -70°C. Powdered liver (1 g) was homogenized at 4°C using a Polytron in 3 ml of homogenisation buffer (4 mM EDTA, 50 mM NaF pH 8.0, 1 mM Na-orthovanadate, 1μM okadaic acid, 0.1% (v/v) 2-mercaptoethanol, with protease inhibitor cocktail). The homogenates were centrifuged at 13000 × g for 10 minutes at 4°C and the supernatant removed and used immediately for Western blot analysis or snap frozen in aliquots at -70°C until needed.

## List of abbreviations

DesCoA: desulfo coenzyme A

HAT: histone acetyl transferase

HDAC: histone deacetylase

IGF: insulin-like growth factor

IRS: insulin receptor substrate

PH: pleckstrin homology

PKB: protein kinase B

PI3K: phosphoinositide 3-kinase

PMA: Phorbol myristic acid

PTB: phosphotyrosine binding domain

TSA: Trichostatin A

## Authors' contributions

CK carried out all of the experimental procedures reported in this study and drafted the manuscript. SRJ conceived of the study, participated in the design of all the experiments and coordinated the study.
